# Consequences of Climate Change-Induced Habitat Conversions on Red Wood Ants in a Central European Mountain: A Case Study

**DOI:** 10.3390/ani10091677

**Published:** 2020-09-17

**Authors:** Orsolya Juhász, Ágnes Fürjes-Mikó, Anna Tenyér, Anna Ágnes Somogyi, Dianne Joy Aguilon, Péter János Kiss, Zoltán Bátori, István Maák

**Affiliations:** 1 Department of Ecology, University of Szeged, Közép fasor 52, H-6726 Szeged, Hungary; ddaguilon@up.edu.ph (D.J.A.); kisspeterjanos003@gmail.com (P.J.K.); zbatory@gmail.com (Z.B.); bikmakk@gmail.com (I.M.); 2 Doctoral School in Biology, Faculty of Science and Informatics, University of Szeged, Közép fasor 52, H-6726 Szeged, Hungary; 3 Department of Forest Protection, NARIC Forest Research Institute, Hegyalja Street 18, H-3232 Mátrafüred, Hungary; mikoa@erti.hu; 4 Department of Physical Geography and Geoinformatics, University of Szeged, Egyetem Street 2-6, H-6722 Szeged, Hungary; a2na9211@gmail.com; 5 Department of Evolutionary Zoology and Human Biology, University of Debrecen, Juhász-Nagy Pál Doctoral School, Egyetem tér 1, H-4032 Debrecen, Hungary; panka.somogyi@gmail.com; 6 Doctoral School of Environmental Sciences, University of Szeged, Rerrich Béla Square 1, H-6720 Szeged, Hungary; 7 Department of Forest Biological Sciences, College of Forestry and Natural Resources, University of the Philippines Los Baños, Laguna 4031, Philippines; 8 Museum and Institute of Zoology, Polish Academy of Sciences, Wilcza Street 64, 00-679 Warsaw, Poland

**Keywords:** clear-cut, oak forest, coniferous forest, colony size, foraging activity, *Formica polyctena*

## Abstract

**Simple Summary:**

The red wood ants are considered to be one of the main ecosystem engineers and keystone species of the habitats in which they exist. Most of the species from this species group inhabit coniferous forests, which, due to the consequences of anthropogenic climate change, are continuously cut down. Therefore, the main habitat of these important species is rapidly disappearing. We investigated the consequences of the absence of conifers (clear-cut area and deciduous forest) on one of the members of this species group, namely *Formica polyctena*. We have found that compared with the mixed-coniferous forest stand, the absence of coniferous species resulted in significant changes in the colony and nest structure of *F. polyctena*. In addition, the colony size was also smaller in these sites. These changes suggest that although *F. polyctena* is able to survive in suboptimal habitats, still their population decline is expected and urges conservation managers to apply necessary action plans for their protection.

**Abstract:**

The consequences of anthropogenic climate change are one of the major concerns of conservation biology. A cascade of negative effects is expected to affect various ecosystems, one of which is Central European coniferous forests and their unique biota. These coniferous forests are the primary habitat of many forest specialist species such as red wood ants. Climate change-induced rising of temperature allows trees to skip winter hibernation, making them more vulnerable to storms that cause wind felling, and in turn, promotes bark beetle infestations that results in unscheduled clear-cuttings. Red wood ants can also be exposed to such habitat changes. We investigated the effects of bark beetle-induced clear-cutting and the absence of coniferous trees on colonies of *Formica polyctena*, including a mixed-coniferous forest as a reference. Our aim was to investigate how these habitat features affect the nest characteristics and nesting habits of *F. polyctena*. Our results indicate that, in the absence of conifers, *F. polyctena* tend to use different alternatives for nest material, colony structure, and food sources. However, the vitality of *F. polyctena* colonies significantly decreased (smaller nest mound volumes). Our study highlights the ecological flexibility of this forest specialist and its potential to survive under extreme conditions.

## 1. Introduction

Global climate change is one of the major threats facing humanity in the 21st century because it contributes to various environmental problems, such as extreme weather conditions (e.g., strong storms and frequent temperature changes) and pest outbreaks [1,2,3]. A cascade of negative effects on various habitats and their biota is expected. For instance, rising temperature allows the trees to skip winter hibernation which makes them more sensitive to unforeseen frost, or snow [3]. Frost-damaged trees are more vulnerable to storms that can cause wind felling, leading to a higher amount of dead wood, which increases the probability of bark beetle (*Ips typographus*) infestations [1,3]. Between 1950 and 2000, half of the biotic damages (e.g., tree decay caused by insect or fungi infection) were caused by bark beetle infestations in European forests [1]. In addition, bark beetle infestations are expected to increase in the 21st century [2], making bark beetles one of the most dangerous pests of coniferous forests in Europe [4,5].

An increase in bark beetle infestations has led forest managers to introduce “unscheduled salvage” cutting (i.e., immediate clear-cut before the forest reaches its cutting age) to save the undamaged timber [2]. In Hungary, the cutting age for trees in coniferous forests is normally between 50 and 70 years (Decree No. 96/2011. (X. 17.) of the Minister of Rural Development). This has a harmful effect on coniferous forest ecosystems, particularly when most of the trees are removed [6]. Likewise, frequent clear-cutting can inhibit the survival or recolonization of coniferous forest specialists. In addition, deciduous tree species are often planted to prevent further bark beetle infestations, however, this method decreases the proportion of older coniferous forests. Deciduous plantations do not have the same environmental conditions as coniferous forests. Therefore, these management practices do not favor the maintenance of coniferous forest specialists [7]. As a result, many plantation forests are becoming more susceptible to the adverse impact of climate change (e.g., monocultures with trees in lines and in even distances are more vulnerable to heavy storms) and increases in other pest infestations [1,3].

Members of the *Formica rufa* group (commonly called red wood ants) are very important keystone species and ecosystem engineers of coniferous forests but decades are usually needed to develop large colonies and fulfil this role [8,9,10]. Investigations in Northern Europe have shown that clear-cuttings hinder the colony development of red wood ants and have a profoundly negative effect on their population survival [11,12,13,14]. Clear-cutting makes the habitat for red wood ants unsuitable and also causes or results in habitat fragmentation. The cleared area is often impenetrable for gynes and workers [7,15,16] and isolation can lead to inbreeding [17]. In addition, clear-cutting negatively influence the strategies of ants to properly exploit food sources (tended aphids) [18]. As a result, aphids on remaining trees produce less and lower quality honeydew which finally leads to starvation and suppressed immune response of ant workers [6,10,18,19]. Ultimately, these effects cause a high mortality rate among the nests in the clear-cut areas [10].

According to our personal observations in Central Europe (e.g., Hungary, Slovakia, and Poland), red wood ants mainly occur in coniferous or mixed coniferous/deciduous forests and are rarely found in deciduous forests, see also [20]. This is the main reason why we have only a limited understanding in terms of the characteristics of the colony structure of red wood ants occurring in deciduous forests [21,22]. It is also unclear whether deciduous plantations can support viable red wood ant colonies for a long term (for many decades).

In this case study, we examine the effects of clear-cutting and the absence of coniferous trees in a deciduous forest on the nest structure and foraging strategies of *Formica polyctena* in the Mátra Mountains (Hungary). We hypothesized that the lack of suitable habitats and proper food sources of *F. polyctena* will result in smaller nest mound volumes, but with an increased number of individual nests and a higher number and/or longer foraging routes. Although our sample size is small, we expect similar results to former studies, i.e., there will be an increase in the number of small nests in the clear-cut site. A high number of small nests, presumably due to nest splitting, leads to an increase in the number of connections among the nests and food sources and can result in shorter lengths of these connections. However, in the deciduous forest site, we expect the lack of conifer needles to lead to alternations in the nest shape and size. The lack of the most preferable food search can affect the foraging routes in the following two ways: (1) optimization processes lead to shorter foraging routes because ants nest close to the best food sources or (2) optimization processes result in longer foraging routes because the limited nesting options are a stronger driver of choice than the closeness of optimal food sources.

## 2. Materials and Methods

### 2.1. Study Sites

**Figure 1:**
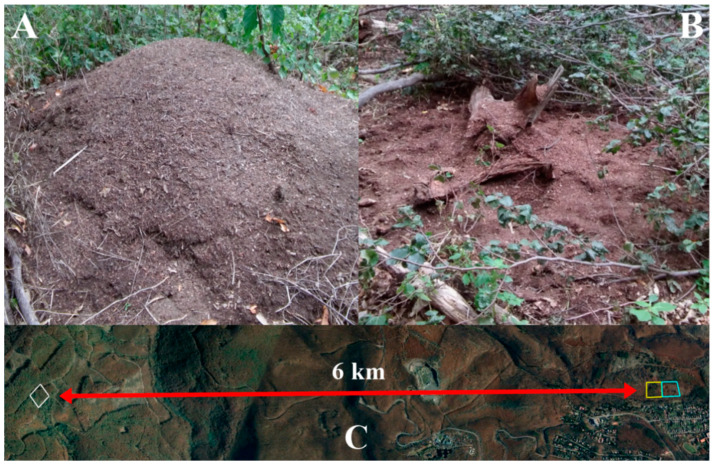
Different nest mound structures in the Mátra Mountains and the sampling sites. (**A**) Half ellipsoid nest shape; (**B**) Changed nest structure in the deciduous site; (**C**) Location of the sampling sites.

Our investigation was performed in the Mátra Mountains (Northern Hungary, 47°49′36.50″ N, 19°58′10.84″ E), at an altitude of 429 m a.s.l., in July of 2017 and 2018. The exposure of the study sites was west to southwest with slopes of 10–15°. We compared the red wood ant nests in three different sites. The first study site was the reference site (RS), a recreational forest covered by a mixed coniferous/deciduous forest with *Pinus nigra*, *Quercus cerris*, and *Q. petraea*, where the age of the trees varied between 2 and 105 years. The second study site was a clear-cut area (Cc), which was once part of a large recreation forest (alongside with our RS) composed of *P. nigra, Q*. *cerris*, and *Q. petraea*. Clear-cutting was done in 2016, because of a bark beetle infestation. The retention trees were mostly *Q. cerris* and *Q. petraea* with a few *P. nigra* (5–10 trees/ha according to the current forestry management practices). Lastly, the third site (Qu hereafter) was a deciduous forest further from the other sites (ca. 6 km by air, Figure 1C) dominated by *Q. petraea*, *Q. cerris*, and *Carpinus betulus.* The trees were between 11 and 116 years old. During the frost period in April 2017, many trees were damaged or uprooted, and serious crown damages were commonly observed on *C. betulus*.

### 2.2. Measurements on the Red Wood Ant Colonies

We used a 150 × 150 m plot (which well represents a red wood ant territory size) [11] to investigate the red wood ant nests in each site. During the assessment, in each site, three people walked in transects 15 m apart from each other (10 transects/site) from edge to edge to identify red wood ant nests. The location of each nest was marked with GPS (GARMIN eTrex^®^ 30, Olathe, KS, USA) and five individuals per nest were collected to identify the species. Abandoned nests were also counted to calculate the nest abandonment ratio.

The size of each nest was determined by measuring the height, the greatest diameter, and the perpendicular diameter. In order to identify changes between the years (2017 and 2018), the measurement was repeated in 2018. The aboveground nest volume (i.e., half ellipsoid, Figure 1A) was determined using the following equation:

(1) $$V=\frac{0.75*\pi *r_{1}*r_{2}*h}{2}$$

where *h* represents the height of the nest mound, and *r_1_* and *r_2_* are the two perpendicular nest radii. This model was used for the Cc and RS. However, for the Qu we developed a new formula because of the slightly different shape of the nests (i.e., not half ellipsoid, Figure 1B). Due to the lack of conifer needles (that allow ants to build their nests with typical shapes) these nests were solely built on larger logs of fallen trees using plant material from the surroundings, such as pieces of *Quercus* acorns, barks, twigs, and leaves. This resulted in substantial changes in the shape of the nests (i.e., more concave shape, where the inhabited parts of the log are in the middle of the nest). Therefore, the following equation was used to describe the shape of the nests:

(2) $$V=\frac{h*\pi }{4}*\frac{4l^{2}-d_{1}*d_{2}}{ln\frac{dl}{d_{1}}+ln\frac{dl}{d_{2}}}$$

where *h* represents the height of the nest mound, *d_1_* and *d_2_* are the two perpendicular nest diameters, and *l* is the radius of the log on which a nest has been built.

To detect changes in the foraging strategies and colony structure of red wood ants within each site, we thoroughly examined every individual nest and mapped the foraging routes belonging to them. We also mapped the trunk trails of the nest’s permanent trail system (which often leads to trees where aphid colonies occur or other long-term food sources) [23]. We also noted routes with diffuse ends (where ants searched for invertebrate prey) and inter-nest connections using a laser distance measurer (SNDWAY SW-T80, measurement accuracy ±2 mm). To assess the characteristics of nest locations, we measured the girth at breast height (1.3 m) of the closest trees in a circle surrounding the nests relevant to their shading (in 360°). In addition, we noted the species of these trees, and the presence of the aphid-tending ants on them (after following their foraging routes and also checking for ants with honeydew-filled gasters) and measured the distance between trees and nests similar to [24,25,26].

### 2.3. Statistical Analyses

Habitat characteristics (e.g., through disturbance, shading, and microclimate) have a strong effect on nest abandonment ratio. To assess the effect of different habitats on the nest abandonment ratio we compared them among the different sites using a GLM (binomial error, maximum likelihood, *N* = 90). In the model, the status of nest status (abandoned or alive) was included as a dependent variable, whereas the plot ID was an explanatory factor. In addition to nest abandonment, habitat characteristics also have a strong effect on nest mound volumes. Therefore, differences among the studied habitats regarding nest mound volumes were analyzed using GLMs (Gaussian error, maximum likelihood, *N* = 60). Separate models were built for the two different years (2017 and 2018). In the models, the nest volumes were included as dependent variables, whereas the plot ID was an explanatory factor. The changes in the nest volumes between the two years (2017 and 2018) were also compared using a paired Wilcoxon test. Analyses were performed including all sites, and separately for each plot. 

The length of foraging routes can be determined by nest volume and can be a trade-off with the number of foraging routes. Therefore, we analyzed the effects of the nest volume and the number of foraging routes on the length of foraging routes using LMM (Gaussian error, maximum likelihood fit, *N* = 40). In the model, the average route length per nest was included as a dependent variable, whereas the mound volume and the number of foraging routes were explanatory factors, with plot ID as a random factor. We also assessed the effect of the habitat type on the length of the different foraging routes found in each colony by comparing them among the studied sites using LMM (Gaussian error, maximum likelihood fit, *N* = 520). In the model, the length of the foraging routes was included as a dependent variable, the plot ID as an explanatory factor, and the colony ID as a random factor. The effects of the number of foraging routes and mound volume on the average length of the foraging routes were also analyzed separately for the different sites using GLMs (Gaussian error, maximum likelihood). In the models, the average length of the foraging routes was included as a dependent variable and the number of foraging routes and mound volume were explanatory variables.

The distance of the food source from the nest mounds can influence the costs of traveling, whereas the girth of the trees can be in relation to the number and size of aphid colonies hosted, therefore, we analyzed the presence of foragers on the nearest trees depending on the girth of the tree and its distance from the ant nest (GLMM, binomial error, maximum likelihood fit, *N* = 285). In the model, the presence or absence of foragers was included as a dependent variable, the girth of the tree and its distance from the ant nest as explanatory factors, and the plot ID as a random factor. The distance of the nearest trees was analyzed using LMM (Gaussian error, maximum likelihood fit, *N* = 285). In the model, the distance of the trees was included as a dependent variable, the plot ID and the girth of the trees as explanatory factors, with the nest ID as a random factor. To assess the potential differences among habitats in terms of available food sources, we also compared the girths of the nearest trees among the sites using LMM (Gaussian error, maximum likelihood fit, *N* = 285). In the model, the girth of the trees was included as a dependent variable, the plot ID as an explanatory variable, and the nest ID as a random factor. 

Nest size can have a close relationship with the vitality of a colony; therefore, we also investigated the effect of different habitat characteristics on the nest volume separately for the different sites using GLM (Gaussian error, maximum likelihood). In the models, the nest volume was included as a dependent variable, the girth of the adjacent trees, the distance between the adjacent trees, the interaction of the two variables, and the number of trees as explanatory variables.

All statistical analyses were carried out in the R Statistical Environment [27]. LMMs and GLMMs were performed using the lmer or glmer function, respectively (lme4 package) [28] and automated model selection with the help of the dredge function (MuMIn package) [29]. GLMs were performed with the glm function and automated model selection with the help of the step AIC function (MASS package) [30]. The lsmeans function of the lsmeans package was used in order to carry out post-hoc sequential comparisons among factor levels when performing GLMs and GLMMs [31]. When it was necessary, variables were log-transformed (nest mound volume, average foraging route length, distance of the nearest trees) for normalization.

We used QGIS 2.1.8 for distance calculations and the visual representation of our data [32]. A vector file was generated based on the GPS location of each nest. Using this vector file, we generated the heat map of the sites. By adding the nest volumes to the vector file and with the help of gradual setting (7 size categories, i.e., Category 1, 0–100 dm^3^; Category 2, 100–200 dm^3^; Category 3, 200–500 dm^3^; Category 4, 500–750 dm^3^; Category 5, 750–1000 dm^3^; Category 6, 1000–1500 dm^3^; and Category 7, 1500–5000 dm^3^), we generated the maps of the nests. We calculated the nearest neighbor index and Z-scores of the studied sites using the Nearest Neighbor Analysis tool.

## 3. Results

*F. polyctena* formed polydomous colonies with interconnected nests in the study area. Different nest abandonment ratios were found in the sites: 45% in the RS, 22% in the Cc, and 11% in the Qu. The nest abandonment ratio in the RS was significantly higher than in the Qu (GLM, *t* = 2.54 and *p* < 0.05). In the other two cases, we did not find any significant difference (*t* < 1.9 and *p* > 0.05).

**Figure 2:**
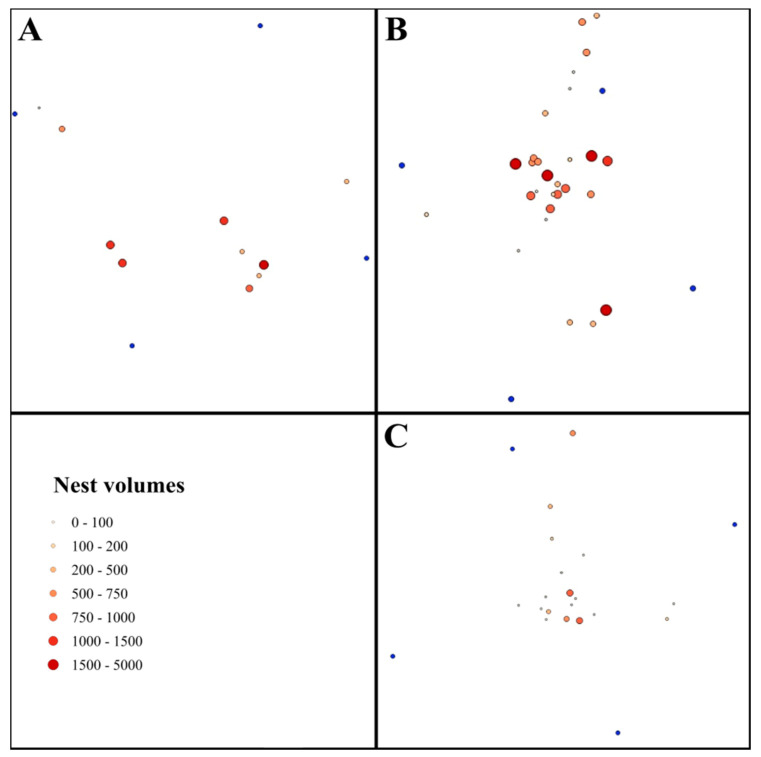
The nest volumes in the studied sites of the Mátra Mountains. (**A**) The reference site; (**B**) The clear-cut site; (**C**) The deciduous site.

**Figure 3:**
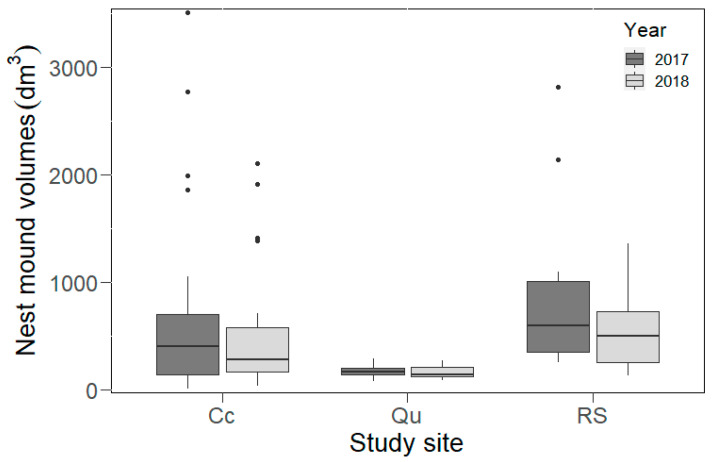
The nest mound volumes (medians, quartiles, range, and outliers) in the studied sites of the Mátra Mountains measured in two consecutive years (2017 and 2018). Cc: clear-cut site; Qu: deciduous site dominated by *Quercus* species; RS: reference site.

In 2017, we found the largest variance in nest mound volumes in the Cc, with the smallest and largest nest mound volumes present in this site (Table 1, Figure 2 and Figure 3). Nest mound volumes were significantly larger in the RS than in the Qu (GLM, *t* = 3.39 and *p* < 0.05) and only marginally larger than in the Cc (*t* = 2.11 and *p* = 0.059). A similar situation was also found in 2018, as the nest mound volumes were significantly larger in the RS (GLM, *t* = 3.12 and *p* < 0.001) and the Cc (*t* = 2.18 and *p* < 0.05, Figure 3) than in the Qu. However, there were no significant differences in the nest mound volumes between the Cc and Qu during either of the years (2017, *t* = −1.77 and *p* > 0.05 or 2018, *t* = −1.38 and *p* = 0.17, Figure 2 and Figure 3). In this year, several nests disappeared in the disturbed habitats (in Cc = 10 and in Qu = 11 nests). A significant decrease in living nest mound volumes was observed in the study area between 2017 and 2018 (Wilcoxon coefficient = 874, *p* = 0.05, Figure 3). Although nest mound volumes decreased in each plot, these changes were significant only in the Cc (Wilcoxon coefficient = 161, *p* = 0.05).

We found the highest nest density in the Cc (21.3 nests/ha) and the lowest in the RS (8 nests/ha). In the Qu, an intermediate nest density (16 nests/ha, Figure 2) was found. According to the nearest neighbor analysis, the nests in each plot showed a more random distribution (RS, NNI = 1.03 and Z-score = 0.16; Cc, NNI = 0.89 and Z-score = −1.18; Qu, NNI = 0.96 and Z-score = −0.31) (Figure 2 and Figure 3).

**Figure 4:**
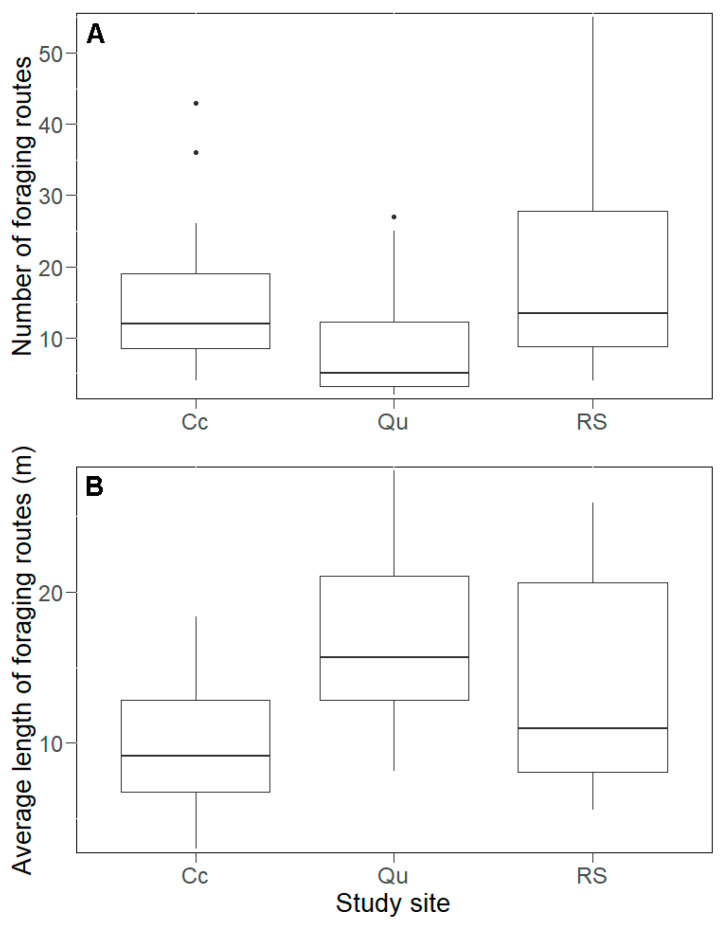
The number (**A**) and average length (**B**) of the foraging routes (medians, quartiles, range, and outliers) found in the study sites of the Mátra Mountains. CC: clear-cut site; Qu: deciduous site dominated by Quercus species; RS: reference site.

We found 590 foraging routes within the study area with an average length of 16 m. The lowest number of foraging routes was in the Qu (124 routes, Table S1 and Figure 4A) but the shortest average length was in the Cc (12 m, Table S1 and Figure 4B). However, the greatest variance in foraging route length was found in the RS, because the shortest (0.62 m) and longest (164 m) foraging routes were present in this plot (Table S1 and Figure 4B). In general, the average foraging route length per nest was positively influenced by the number of foraging routes (LMM, *t* = 7.65 and *p* < 0.001), but not by the nest volume (*t* = 0.41 and *p* = 0.68). The length of the foraging routes was significantly shorter in the Qu compared with the Cc (*t* = −2.91 and *p* = 0.01, Figure 4B). The other comparisons gave no significant differences (*t* < 0.71 and *p* > 0.05, Figure 4B). Taken separately for each plot, we found a positive correlation between the length and the number of foraging routes (GLM, RS: *t* = 6.24 and *p* < 0.01; Cc: *t* = 7.93 and *p* < 0.001; and Qu: *t* = 4.71 and *p* < 0.001). The mound volume did not have a significant effect on the average length of the foraging routes (*t* = 1.76 and *p* > 0.05).

The characteristics of the nest locations of the different sites are presented in Table S2 and Figure S1. In general, the girth of trees had a positive effect (GLMM, z = 5.64 and *p* < 0.01), whereas the distance of trees from the nests had a negative effect (z = −3.93 and *p* < 0.01) on the presence of aphid tending ants. On the one hand, the distance of the nearest trees from the nests was positively influenced by the girth of trees (LMM, t = 3.62 and *p* < 0.001), however, there were no differences among the different sites in this respect (t < 0.92 and *p* > 0.05). On the other hand, the girth of trees was significantly larger in the Qu than in the other two sites (RS, t = −7.05 and *p* < 0.001; CC, t = −8.321 and *p* < 0.001). The difference was only marginally significant (t = 2.39 and *p* = 0.06) between the RS and Cc. In the Qu, both the girth of trees (GLM, t = 2.93 and *p* = 0.01) and the distance of nearest trees (t = 2.8 and *p* < 0.05) had positive effects on the nest mound volume, and the interaction of these two variables had a negative effect on the nest volume (t = −2.69 and *p* < 0.05). In the other two sites, neither of the variables had a significant effect (t < 1.24 and *p* > 0.05).

## 4. Discussion

Our results are consistent with the expected effects of clear-cutting and the absence of coniferous trees on nest mound volumes and foraging habits of the polydomous red wood ant colony systems. Findings from our study indicated a larger variance in nest mound volumes in the clear-cut area which were characterized by slightly smaller nests as compared with the nests found in the reference site (i.e., mixed coniferous/deciduous forest). Ants in the clear-cut areas used the shortest foraging routes. However, the deciduous forest patch resulted in a very different nest structure and foraging habits, having the smallest sized nests and longest foraging routes. This supports our second hypothesis about the effects of the deciduous forest environment which stated that availability and location of suitable nesting sites could be a stronger determining factor for red wood ants than the distance from food sources that led to longer foraging routes. Altogether, we found polydomous systems with random nest distribution in each plot that could be the result of the spreading strategy of *F. polyctena*. Polydomous colonies are spread by nest splitting which can lead to the uniform coverage of the territory [33] and are also influenced by the location of appropriate nesting sites. This distribution is in accordance with the findings of Tsikas et al. [25] for *F. lugubris* in the Rhodope Mountains (Greece). However, since we investigated only one repetition per treatment, the possibilities for generalization of the results should be handled cautiously.

We found a high nest abandonment ratio (45%) in the reference site, which was similar to that found 4–5 years after clear-cutting in Finland [11,12,13]. However, in our case, this pattern could be related to the lack of appropriate insolation and disturbance regimes [34,35,36,37,38]. Our results showed that the nests were situated away from trees with larger girth which could be due to the shading effect of the large trees. Thus, there may be a correlation between the distance of foraging routes and the girth of trees. New nests can be established in areas with a sufficient amount of sunlight [34,36,37]. In our reference site, appropriate light conditions were found only at the edge of pathways that were kept clear for the tourists, but smaller nests did not tolerate larger disturbances (e.g., high trampling intensity) [35,38]. As a result, new nests could be established only in less visited and trampled areas. In addition, rapid shrub encroachment can also lead to high nest turnover. Nonetheless, old and large colonies can persist under these disturbances, close to the most visited tourist sites, or in overgrown and shaded areas [34,36,37,38]. Abandoned nests were usually close to larger nests, possibly as the result of nest relocations over time (i.e., finding better conditions for the growing colonies). The abandoned nests were still recognizable, presumably due to the slower degradation rate of the pine needles in the nest material.

Although the reference site had the smallest number of nests, it showed considerable variation in nest size distribution and foraging route length. The main reason for this was that the food availability and quality in this plot were close to a preferred habitat of red wood ants with many coniferous trees. The presence of larger and closer conifer trees support ant populations by providing proper food sources and allow them to visit aphids. In general, the nest volumes did not change significantly between the years, and all the nests were present in both years of the study.

Nest abandonment ratio was 22% in the clear-cut plot, which was lower than that found in 4–5 years old cuttings in Finland [11,12,13]. It is important to note that our clear-cut plot was sampled one year after the disturbance, whereas ant colonies in Finland had more time to react [11,12,13,39]. However, the nest mound volumes decreased between the years, indicating that clear-cutting had a significant effect on red wood ants, even after two years of clear-cutting. Presumably, the rate of abandonment should increase in the future as many nests disappeared from 2017 to 2018. The clear-cut area provided habitat for the largest number of nests, and it showed the largest variation in nest mound volumes (from the smallest to the largest in the study area), which reflected the age of colonies [40]. This large variation indicated that very young and old nests were present in this area. This was presumably due to the fact that many large and old colonies survived under the protection of remaining shrubs and deciduous trees and whereas more exposed larger nests were split into several small ones in order to reach the nearest forest edge [10,11,12,13,39,41]. The newly established small nests were mostly found in quite exposed areas, on the trunks of recently cut trees. This could lead to the desiccation of the small nests [42] resulting in a higher mortality rate, which is already elevated due to a reduced capacity to efficiently defend against competitors, predators, and parasites, as well as lowered thermoregulation capability [10]. Some small nests still occurred in “unoccupied” and less exposed sites under shrubs or scattered trees. Ants using this strategy could stay closer to the remaining aphid colonies, allowing them to compensate for the lack of enough food sources, which can be one of the main problems of the nests in clear-cut areas [6,11,12,13]. Ants also visited larger trees that could support a larger number of aphid colonies. 

As predicted, our findings are in accordance with previous studies which have shown that clear-cutting had a strong effect on the colony size of red wood ants, resulting in smaller nest mound volumes compared to a reference plot [10,11,12,13]. We also found the highest number of foraging routes and the shortest average length in the clear-cut plot. The increased number of connections among the newly established nests and mother nests increased the number of foraging routes and decreased their distances, as also shown in former studies [10,11,12,13,39]. After clear-cutting, the quality and quantity of aphid honeydew decreased [6]. This could lead to colony starvation and to a decrease in the size of workers [11,12,13].

The nest abandonment ratio was the lowest (11%) in the deciduous plot, similar to the ratio (10%) found for the managed part of the Białowieża Primaveral Forest (Poland) [20]. We also found the smallest nest size in the deciduous plot (tree size had a negative effect on the nest size). This is in contrast with other studies, which have shown that nest size grows with increasing shade [20,43]. As a replacement for the absence of conifer needles, the ants use fallen trees to strengthen their nest. Without the needles, the nests are lower but longer alongside the fallen tree trunks. This is in agreement with other studies which indicated that the complete absence of conifers caused abnormalities in wood ant colonies such as changed nest size structure and distribution [21,22]. For instance, Kristiansen and Amelung [21] found that *F. polytena* built below-ground nests in an oak forest. In addition, Dolek et al. [22] found that *F. polyctena* associated with semi-open forest stands if the forest was primarily composed of oak species.

The fewest but longest foraging routes were found in the deciduous plot with a preference for *Q. cerris* trees (ca. 90%). This is in contrast with the findings in *Formica lugubris* which does not show a preference for any tree species in mixed coniferous/deciduous forests where both *Pinus sylvestris* and *Quercus* spp. are available [26]. However, other studies have found that *F. lugubris* used *Quercus* trees as the main food resource [44]. Our findings suggest that *Q. cerris* has great potential to be a sufficient substitute to replace conifers for red wood ants. However, ants try to find the best aphid-infested trees, and therefore establish longer routes to find them. We found a negative correlation between the number of foraging routes and their length. This was the consequence of the nests’ cost-efficiency optimization [45]. The limited number of workers cannot maintain unlimited foraging routes. Therefore, the workers have to optimize their choice between the number of routes and the quality of the honeydew of the visited trees.

Food searching behavior of the red wood ants is primarily related to the distribution of aphids on conifers [6,34,46,47]. In the absence of enough coniferous trees, ants used *Q. cerris* trees (42% of the searching trees) to fulfil their needs. We strongly suspect that the decreased nest mound volumes in the deciduous forest are in relation to the insufficient food sources provided by aphids on deciduous trees because this forest is slightly older than the others, and older forests can support larger red wood ant colonies [6]. In addition, many nests disappeared between the years. Robinson et al. [44] showed that deciduous trees could also provide important resources for *Formica lugubris*, but the use of such resources was lower than their abundance predicted. In addition, if conifers are also present, ants use them in a disproportionately large amount [44]. This is in line with the findings of Sondej et al. [20], who reported that deciduous forests in Poland usually did not provide a suitable habitat for red wood ants. Another possible explanation for these patterns is the lack of conifer resin in the deciduous plot. Red wood ants collect and use the pine resin actively to disinfect the nest material [48], and therefore the lack of conifer resin can lead to the decline of populations. 

In this case study, we have taken the first step to understand how clear-cutting and the absence of conifers could affect nest size and foraging strategies of red wood ants in Central European managed forests. These disturbances will become more common in the future because the frequency of bark beetle infestation and wind felling will increase due to the effects of anthropogenic climate change [3]. Although the sample size was small, our results indicate that different disturbances cause different responses within red wood ant colonies such as nest splitting in clear-cuts and the use of fallen trees for nesting in a deciduous forest. Their foraging behavior can also change in response to a changing environment, from pine-feeding aphids to oak-feeding aphids. It is also possible that they use tannin from *Q. cerris* trees to replace the missing antimicrobial potential of pine resin. Although we cannot rule out other mechanisms (such as the effect of site differences) due to the small sample size, this work provides the first step to understand the consequences of conifer loss on this ecologically important species group, and we encourage myrmecologist to conduct further investigations with larger sample sizes to clarify these aspects. 

The smaller nest mound volumes observed in the studied sites without coniferous trees could lead to decreased surviving chances of red wood ants in the changed habitats. Although they seem to be ecologically flexible and also can survive under extreme conditions [39,49,50,51,52], the disappearance of many nests in the disturbed habitats gives concern and calls for action from conservation managers. However, further investigations on a larger scale and with a larger sample size are needed to answer the still unresolved question, “How long can red wood ants (e.g., *F. polyctena*) survive in a rapidly changing environment?” The results of our case study indicate that nest relocation would be advantageous before clear-cuttings and it would be important to plant mixed forest stands instead of monocultures in areas where the climate is suitable for native coniferous tree species. These activities could help to maintain viable populations of this ecologically significant forest specialist species in the long term.

## 5. Conclusions

Our results highlight that climate changed-induced habitat loss negatively affects red wood ants. The decrease of the colony size (nest volume) is irrespective of the cause of loss (e.g., clear-cut or deciduous forest without any conifers). However, red wood ants try to adapt to suboptimal conditions as follows: (1) they use alternative nesting strategies (nest splitting after clear-cutting and fallen logs in deciduous forest), (2) they use alternative food sources (aphids on *Q. cerris* trees), and (3) they optimize their searching strategies (shorter but increased number of searching routes after clear-cutting and longer but fewer routes in deciduous forest). Despite these adaptations, the decreased colony size makes these colonies more vulnerable to environmental conditions, predators, and parasites. Therefore, further research is needed to develop new conservation management tactics to protect this ecologically significant species group.

## Figures and Tables

**Table 1:** Nest mound characteristics of *F. polyctena* colonies in the Mátra Mountains.

	RS	Cc	Qu	Study Area
Number of nests	22	41	27	90
Number of living nests 2017	12	32	24	68
Number of living nests 2018	12	22	13	47
Min. nests mound volumes 2017	250 dm^3^	7.9 dm^3^	82 dm^3^	7.9 dm^3^
Max. nests mound volumes 2017	2812 dm^3^	3506 dm^3^	288 dm^3^	3506 dm^3^
Average nests mound volumes 2017	895 dm^3^	638 dm^3^	167 dm^3^	542 dm^3^
Average nests mound volumes 2018	562 dm^3^	538 dm^3^	163 dm^3^	440 dm^3^

## Supplementary Materials

The following are available online at https://www.mdpi.com/2076-2615/10/9/1677/s1, Figure S1 Heat map of the quadrates from the different forest sites in Mátra Mountains. The blue spots represent the edges of our sampling quadrates. (A) is the reference site; (B) is the clear-cut site; (C) is the deciduous site, Table S1: The searching habits of the red wood ant colonies in Mátra Mountains, Table S2 The characteristics of the nearby trees of red wood ant nests in Mátra Mountains.
