# Supplementary material for: Consequences of Climate Change-Induced Habitat Conversions on Red Wood Ants in a Central European Mountain: A Case Study
Source: Animals (Basel). 2020 Sep 17;10(9):1677. doi: 10.3390/ani10091677 (PMC7552266; doi:10.3390/ani10091677)
Supplement: Supplementary file 1 [file animals-10-01677-s001.pdf]

Table S1 The searching habits of the red wood ant colonies in Mátra Mountains

Nest number	Number of routes	Average length (m)	Min. length (m)	Max. length (m)	*Quercus cerris*
Cc1	34	18.62	3.03	31.38	30
Cc2	9	5.13	1.78	14.7	8
Cc3	4	5.75	2.08	7.64	n/a
Cc4	17	12.73	3.69	23.52	15
Cc5	9	6.71	2.6	16.13	3
Cc6	22	13.42	2.15	24.81	19
Cc7	8	2.57	2.44	5.22	n/a
Cc8	15	11.13	1.63	32.6	9
Cc9	12	12.2	7.34	19.01	8
Cc10	6	7.06	3.48	11.82	4
Cc11	5	2.68	1.59	4.01	n/a
Cc12	19	10.42	2.54	22.09	16
Cc13	4	8.19	4.61	12.99	2
Cc14	9	5.6	2.76	9.71	6
Cc15	12	8.16	4.22	15.32	11
Cc16	9	3.2	1.5	5.2	0
Cc17	5	4.48	2.27	9.64	0
Cc18	23	13.17	2.16	31.42	16
Cc19	43	15.08	1.19	25.64	38
Rs1	53	25.97	5.2	69.6	n/a
Rs2	11	8.96	5.21	14.58	n/a
Rs3	7	7.32	2.29	11.61	n/a
Rs4	48	20.31	0.62	48.36	40
Rs5	20	21.9	5.46	44.8	12
Rs6	7	7.14	2.74	16.11	0
Rs7	1	1.76	1.76	1.76	0
Rs8	16	12.93	3.91	23.91	16
Qu1	4	7.27	5.16	8.43	n/a
Qu2	19	24.41	2.21	48.2	n/a
Qu3	9	19.26	3.21	45.04	n/a
Qu4	2	14.48	6.29	22.66	n/a
Qu5	23	27.7	1.82	49.15	n/a
Qu6	2	6.6	3.05	10.15	n/a
Qu7	2	9.36	9	9.7	n/a
Qu8	14	21.39	6.1	36.14	n/a
Qu9	2	8.66	3.47	13.86	n/a
Qu10	4	12.58	3.58	28.73	n/a
Qu11	2	15.32	3.57	27.08	n/a
Qu12	5	10.61	3.72	22.05	2
Qu13	4	18.57	10.21	28.58	n/a
Qu14	1	18.9	18.9	18.9	1

Table S2 The characteristics of the nearby trees of red wood ant nests in Mátra Mountains

Nest number	Nearby trees (pc.)	Average distance (m)	Min. distance (m)	Max. distance (m)	Average perimeter (cm)	Min. perimeter (cm)	Max. perimeter (cm)	Trees with ants	*Q. cerris* with ant visitors	Pillar trees
Cc1	6	4.94	3.03	8.03	89.22	31	147	6	4	0
Cc2	6	2.81	1.77	4.44	87.5	39.5	123	6	5	0
Cc3	12	4.62	0	8.23	60.62	38	94	7	7	3
Cc4	4	3.4	0	5.18	52	25.5	75.5	4	3	1
Cc5	16	5.7	2.26	16.13	54.75	9	147	9	6	0
Cc6	14	6.06	2.15	12.05	64.28	11	128	7	6	0
Cc7	9	3.56	2.44	5.22	29.55	12	45	8	3	0
Cc8	6	4.07	1.63	6.2	55.3	4.8	108	3	2	0
Cc9	1	0	0	0	13	13	13	1	1	1
Cc10	5	1.65	0	3.48	29.13	1	125.4	5	0	1
Cc12	10	6.16	2.54	9.15	53.75	15	102	8	7	0
Cc13	5	3.3	0.85	5.8	40.8	17	66	1	0	0
Cc14	9	5.05	2.76	7.71	56.11	18	93.5	9	6	0
Cc15	10	8.35	3.5	13.1	84.63	29	251.32	5	4	0
Cc16	4	1.39	0	2.5	10.52	7.85	14.13	4	0	1
Cc17	2	2.64	2.27	3	33	26	40	2	0	0
Cc18	4	2.41	0	4.45	90.33	40.8	150	4	2	1
Cc19	5	3.5	1.19	7.4	114.46	70.5	251.32	5	4	0
Rs1	9	2.64	0.96	5.56	13.82	1.5	59	1	0	0
Rs2	6	3.37	2.8	4	47.56	17.9	114.7	2	0	0
Rs3	7	2.66	2.33	3.42	37.64	10	119	2	1	0
Rs4	7	1.95	0	3.89	33.34	7	99.4	5	1	1
Rs5	11	2.44	1.35	5.46	28.86	6.7	113	1	0	0
Rs6	14	2.17	0	3.55	44.42	8	128	10	0	3
Rs7	6	2.01	1.55	2.63	62.63	23.5	131	1	0	0
Rs8	6	4.88	2.64	6.64	56.45	23.9	109	4	1	0
Rs9	4	1.84	0	3.57	47.8	18.7	81	0	0	1
Qu1	10	6.26	4.15	8.43	134.38	100	166.5	4	4	0
Qu2	5	3.41	2.16	6.68	92.6	72.5	123.8	1	1	0
Qu3	8	5.15	2.68	8.59	119.92	63	145.5	2	2	0
Qu4	8	4.73	3.15	6.86	108.14	50.5	163.9	0	0	0
Qu5	6	4.35	1.82	5.81	105.71	50.3	164.5	3	3	0
Qu6	10	5.07	3.05	7.59	85.02	39.4	191.6	1	1	0
Qu7	3	3.13	2.12	3.76	118.45	90.7	147.65	0	0	0
Qu8	6	7.3	1.91	11.19	122.37	78	190	2	2	0
Qu9	8	4.83	2.72	7.16	106.76	76.7	144.5	1	1	0
Qu10	5	5.88	1.56	10.01	82.8	11.2	156.5	1	1	0
Qu11	6	4.28	3.27	6.96	125.45	70.5	167.5	1	1	0
Qu12	7	4.66	1.84	7.02	126.27	65	145	2	2	0
Qu13	5	5.67	2.86	9.64	118.27	65.4	164.3	0	0	0

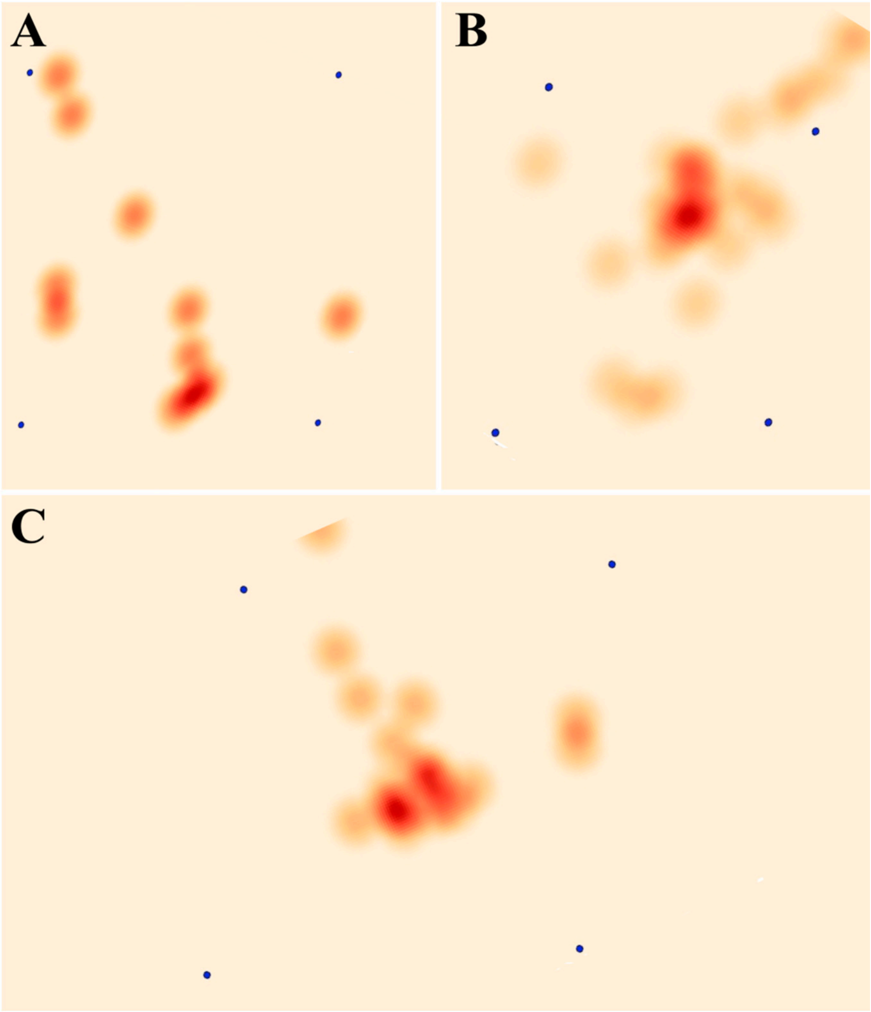

Figure S1 Heat map of the quadrates from the different forest sites in Mátra Mountains. The blue spots represent the edges of our sampling quadrates. (A) is the reference site; (B) is the clear-cut site; (C) is the deciduous site
